# Physicochemical Characteristics, Antioxidant Activities, and Aroma Compound Analysis of Seven Peach Cultivars (*Prunus persica* L. *Batsch*) in Shihezi, Xinjiang

**DOI:** 10.3390/foods11192944

**Published:** 2022-09-20

**Authors:** Huimin Wu, Youyou Xu, Huan Wang, Yuanyuan Miao, Chunyan Li, Ruirui Zhao, Xuewei Shi, Bin Wang

**Affiliations:** Food College, Shihezi University, Shihezi 832000, China

**Keywords:** antioxidant activity, aroma characteristics, physicochemical characteristics, peach

## Abstract

Peaches are tasty and juicy, with a unique flavor. The flavors of peaches always vary with cultivars. To investigate the physicochemical and aroma characteristics of peaches, the sugars, organic acids, total flavonoids, phenols, antioxidant activities, and aroma compounds of seven peach cultivars in Xinjiang were determined using high-performance liquid chromatography (HPLC) and headspace solid-phase microextraction coupled with gas chromatography–mass spectrometry (HS-SPME–GC–MS). The results showed that sucrose (59.83 to 87.34%), malic acid (32.41 to 59.14%), and chlorogenic acid (10.43 to 45.50%) were the dominant sugar, organic acid, and phenolic compound in peaches, respectively. The antioxidant activity varied between 147.81 and 394.55 μmol TEs/100 g. The analysis of the aroma structure of peaches found that the volatile composition of peaches was relatively consistent, though the concentration of total aroma and certain separate compounds were different between cultivars. Meanwhile, the aroma fingerprint of the peaches consisted of hexyl acetate, cis-3-hexenyl acetate, γ-decalactone, n-hexanal, 2-hexenal, nonanal, decanal benzaldehyde and 6-pentylpyran-2-one, providing a clear green, sweet, floral, and fruity odor. These results provide complete information on the physicochemical properties, functional ingredients and aroma of the peaches.

## 1. Introduction

The peach (*Prunus persica* L. *Batsch*), which belongs to the subfamily Prunoideae of the family Rosaceae, is one of the economically important fruits in the world [1]. Peaches were widely cultivated in China as early as 3000 years ago and gradually spread to other temperate regions of Asia [2]. Botanically, peaches are known as drupes or “stone fruits”, such as mangoes, cherries, lychee, and apricots, in which a hard core is surrounded by an outer pulp part [3]. The peach is becoming increasingly popular among consumers worldwide due to its visual appeal, nutritional value, and distinct flavor characteristics such as high juiciness, sweetness, aroma, and soft texture for consumption [4,5]. There are over 3000 peach cultivars in the world [6], with approximately 800 of them grown in China, such as the flat peach, honey peach, nectarine, yellow peach and shoutao. Among them, the flat peach has become one of the famous distinctive fruits in Xinjiang, China, on account of its excellent taste and flavor.

The peach contains a high concentration of vitamins (including vitamin A, vitamin B, and vitamin C) and trace elements (calcium and phosphorus) [7]. Especially, the peach contains more than 1 mg/g of iron, ranked second among fruits [8]. Peaches contain a variety of phenolic compounds, such as flavan-3-ols, flavonols, and anthocyanins, which endue peaches with a high antioxidant capacity that is beneficial to our health [9]. Phenols in the peach are recognized as excellent sources of natural antioxidants [10]. Kono et al. [11] believed that the peach pulp extract possesses cardiovascular protective effects. Phenolic compounds in fruit also play a crucial part in desirable qualities (taste, color and antioxidant) [12] and undesirable ones (astringency and bitterness) [13].

In addition to a high nutritional value, a juicy and tasty peach also possesses a wonderful flavor [14]. Aroma compounds significantly contribute to the flavor and eating attractiveness of fruit, thus volatile compounds are important and necessary components of fruits [15]. As headspace solid-phase micro-extraction coupled with gas chromatography-mass spectrometry (HS-SPME–GC–MS) technology has been generally applied to extract, isolate, detect and identify volatile compounds, more than 100 volatiles have been identified, based on compound structures that could be classified as aldehydes, alcohols, terpenoids, esters, and lactones [5,16,17,18]. It has been found that the most abundant are C6 compounds, esters and lactones in the peach, particularly γ-decalactone and δ-decalactone [19]. According to Visai and Vanoli [20], C6 aldehydes and alcohols such as nonanal and hexanol gave maturing peaches a “green and grassy” aroma while lactones like γ-decalactone and δ-decalactone were substantially connected with a “peach-like” odor.

Peach flavor and quality always varies with the location, climate, variety and growth period [21]. Xinjiang possesses plenty of sunshine, a great difference in temperature between day and night, and abundant light and heat resources, which makes Xinjiang suitable to grow fruits, such as various peaches [22]. Though the physicochemical properties and aroma characteristics have been evaluated in various peaches, the aroma characteristics of peaches in Xinjiang are still unclear. To investigate the specific flavors of peaches, sugars, organic acids, antioxidant activity, total flavonoids, phenolic compounds, and aroma compounds were determined based on high-performance liquid chromatography (HPLC) and HS-SPME–GC–MS technology. Moreover, the aroma fingerprint of the peach was constructed based on a multivariate analysis of volatile compounds. The results will provide much more useful information for future breeding to improve fruit quality by regulating phenolic content or aroma characteristics.

## 2. Materials and Methods

### 2.1. Raw Materials

**Figure 1:**
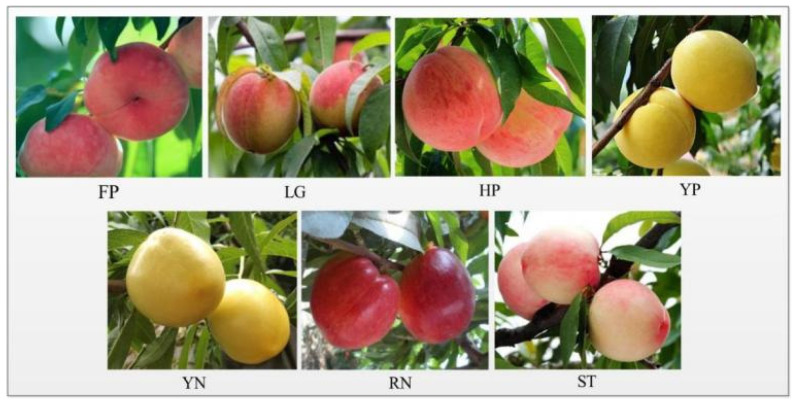
Photos of peaches. Flat peach (FP), liguang peach (LG), honey peach (HP), yellow peach (YP), yellow nectarine (YN), red nectarine (RN) and shoutao (ST) from shihezi, Xinjiang. Every batch of samples (60) was taken from the upper, middle, and lower positions of each tree.

A total of 7 cultivars of peaches were harvested from shihezi, Xinjiang, China, including the flat peach (FP), liguang peach (LG), honey peach (HP), yellow peach (YP), yellow nectarine (YN), red nectarine (RN) and shoutao (ST) (Figure 1). A total of 420 peach fruits and 60 for each sample were taken from different positions of 5–6 (upper, middle and lower) trees (5–15 years old). Ripe peaches that had not been damaged or infected by fungi were harvested based on color, firmness, aroma and the grower’s judgment. After the harvest, peaches were randomly divided into three replicates. Peaches were immediately washed with distilled water within 2 h, and the peach surface was wiped dry with gauze, then enucleated and packaged in an airtight polyethylene bag and stored at −4 °C until analysis.

### 2.2. Chemicals

The standard products with the concentration of 99%, including sugars (sucrose, sorbitol, fructose, glucose), organic acids (quinic acid, malic acid, citric acid, succinic acid), phenolic compounds (*p*-coumaric, procyanidins b, neochlorogenic acid, catechinic, vanillic acid, chlorogenic acid, epicatechin, rutin, quercetin, kaempferol) and 3-octanol were obtained from Yuanye Biotechnology Co., LTD (Shanghai, China). The 2,2′-azinobis (3-ethylbenzothiazoline-6-sulphonate) (ABTS^+^), ferric reducing antioxidant power (FRAP), 2,2-diphenyl-1-picrylhydrazyl (DPPH), and cupric reducing antioxidant capacity (CUPRAC) reagents were purchased from Beifang Tianyi Chemical Reagents Factory (Tianjin, China). All other chemicals were of analytical grade.

### 2.3. Soluble Solids Content (SSC) and Titratable Acidity (TA) Determination

The determination of soluble solids content and titratable acidity was based on juice squeezed from 10 peaches randomly selected from each cultivar. Soluble solids content was determined by sugar refractometer (Atago PR-101R, China) [23]. Titratable acidity was measured by using titrated NaOH (0.1 mol/L) and indicated using a percentage of malate (%) [24]. Three replicates were used in each group.

### 2.4. Total Phenolic and Total Flavonoid Content Determination

Determination of total phenolic content (TPC) referred to the Folin–Ciocalteu method [25]. In order to fully extract the phenolic content, peaches were pulverized to powder with liquid nitrogen. Next, 2.5 g powder with 12.5 mL methanol aqueous solution (80%; *v*/*v*) underwent ultrasonic extraction two times for 30 min at 35 °C, then centrifugation was performed at 10,000 rmp for 20 min. After extraction, the supernatants were mixed to obtain the polyphenolic extract. For the determination, 1 mL of polyphenolic extract mixed with 1 mL Folin–Ciocalteu reagent (5 times diluted) were placed in a tube, left to stand for 5 min, then added to 4 mL Na_2_CO_3_ solution (7.5%; *w*/*w*) and reacted for 60 min at 25 °C. At 765 nm, the absorbance was determined using a spectrophotometer. The gallic acid equivalent per 100 g of dry mass (mg GAE/100 g) was used to express the TPC content.

Total flavonoids content (TFC) was detected according to Cao et al. [26]. First, 1 mL of polyphenolic extract and 0.1 mL NaNO_2_ solution (5%; *w*/*w*) were added to the tube and held at 25 °C for 5 min, then added to 0.15 mL of AlCl_3_·6H_2_O (10%; *w*/*w*) solution. After reacting for 5 min, 0.6 mL NaOH (0.1 mol/L) solution was added to fix the extract at a volume of 3 mL. Spectrophotometer measurements of the absorbance were obtained at 510 nm. The standard used was rutin (RE), and the results were displayed as mg of RE per 100 g of dry mass (mg RE/100 g).

### 2.5. Determination of Sugars and Organic Acids by HPLC

The method of sugar extraction referred to Orazem et al. [27], and determinations were performed as described in Bae et al. [28]. First, the enucleated peaches were pulverized and 1.0 g of peach power was added to a centrifuge tube, then mixed with 20 mL distilled water. Samples were blended using a vortex instrument for 1 min, followed by ultrasound extraction for 30 min at 30 °C, then centrifugation at 10,000 rpm for 15 min at 4 °C. The vortex extraction was repeated twice, and the supernatants were combined into a sugar extract, then fixed at a volume of 40 mL, and finally filtered through a 0.22 μm water-filter film. Samples were analyzed using HPLC technology. Acetonitrile and water (80:20; *v*/*v*) served as the mobile phase in the chromatographic separation of sugars at a flow rate of 0.8 mL/min, along with an XB ridge amide column (5 μm, 4.6 mm × 250 mm). The injection volume was 10 μL and the column temperature was 30 °C. An RID-10 differential detector was used to find the eluted peaks.

The organic-acid-extraction method was performed according to Orazem et al. [29] and analyzed according to Aubert et al. [30] with some modifications. Samples were analyzed using HPLC technology. At a flow rate of 0.8 mL/min, 3% methanol (A) and ultrapure water (B) were used in the chromatographic separation process for organic acid detection. The samples were injected into a Waters C 18 column (5 μm, 4.6 mm × 150 mm; Waters, USA) for analysis. The injection had a 10 μL volume and the chromatographic column was kept at 30 °C. A PAD detector (Waters, Palo Alto, CA, USA) was used to detect organic acids, which were found at a wavelength of 210 nm. The concentration of sugars and organic acids in peaches were calculated by the standard curve of the standard products.

### 2.6. Phenolic Compound Determination

The extraction of phenolic compounds was adjusted appropriately according to Chang et al. [31]. The preprocessed peach powder (3 g) was added to a centrifuge tube. Then, 15 mL of a methanol–ultrapure water mixture (80%; *v*/*v*) was added, mixed with vortex instrument for 1 min, and sonicated at 25 °C for 40 min, then the mixture was centrifuged at 10,000 rpm for 15 min. This was repeated twice and the supernatants were merged. Finally, the extract was concentrated at 30 °C using a rotary steam instrument, and the extract was adjusted to a volume of 30 mL using methanol and ultrapure water.

The phenolic compounds were detected as described in Juaniz et al. [32], applying HPLC with a Waters C18 column (5 m, 4.6 mm × 150 mm; Waters, USA). The eluting solvent was methanol (A) and acetic acid aqueous solution (1%; B). Column temperature: 30 °C; injection volume: 10 μL; and the flow rate: 0.8 mL/min. The elution procedure: 5% A at 0 min, 40% A at 35 min, 95% A at 55 min, and 5% A at 60 min. PAD detector wavelength: coumaric acid, proanthocyanidin, catechin, vanilloid acid, and epicatechin were measured at 280 nm, chlorogenic acid and neochlorogenic acid at 330 nm, and rutin and quercetin at 360 nm. The content of phenolic compounds was calculated by the standard curve of the standard product.

### 2.7. In Vitro Antioxidant Activity Determination

The ABTS^+^-radical-scavenging capacity was analyzed according to Re et al. with some modification [33]. The extract preparation was the same as described in 2.6. First, 0.1 mL polyphenolic extract was mixed with 3.9 mL ABTS solution under dark conditions and reacted for 8 min, then the absorbance was measured at 732 nm. The method of DPPH-free-radical-scavenging activity referred to Gomathi et al. [34], and was slightly modified. First, 0.1 mL polyphenolic extract was mixed with 3.9 mL DPPH solution under dark conditions and reacted for 30 min, then the absorbance was measured at 517 nm. The analysis of CUPRAC and FRAP referred to previous method [35,36]. The results were shown as μmol Trolox equivalents in 100 g of dry mass (μmol TEs/100 g).

### 2.8. Aroma Compound Determination

The aroma compounds were analyzed by the HS-SPME–GC–MS method. Sample preparation and extraction conditions referred to Gong et al. [37]. First, 4.5 g of peach powder and 0.5 g of saturated sodiun chloride were added to a 20 mL centrifuge tube, followed by 2 μL of 3-octanol solution (330 μg/kg; internal standard). The mixed solution was equilibrated at 45 °C for 15 min and extracted at the same temperature for 45 min using an SPME fiber (57329-U; Supelco Inc. Bellefonte, PA, USA) coated with divinylbenzene/ carboxen/ polydimethylsiloxane (DVB/CAR/PDMS, 50/30 μm). After extraction, the fiber was inserted into the gas-chromatography (GC) injector. The desorbed volatiles were managed in split-less mode at 250 °C for 2 h.

Volatile compounds were analyzed using Agilent 7000D-GCMS detector (Agilent Technologies Inc, Palo Alto, CA, USA) combined with an HP-Innowax column (30 m × 0.25 mm × 0.25 µm), with helium serving as the carrier gas at a continuous flow of 1 mL/min. The injection port temperature was 230 °C. The GC device was set at a starting temperature of 40 °C for 5 min, followed by ramps of 4 °C/min up to 86 °C for 5 min, 1.5 °C/min up to 90 °C, 5 °C/min up to 180 °C for 3 min, and finally a ramp of 10 °C/min up to 230 °C, which was held for 5 min. With an ionization source temperature of 270 °C, an electron impact (EI) ionization energy of 70 eV, and a scanning rate of 5 scans per second, mass spectra were captured in the m/z range of 35 to 350.

The National Institute of Standards and Technology (NIST 98) and Wiley 6 mass spectral libraries were used to identify volatile chemicals by comparing their mass spectra. Additionally, semi-quantification was employed to calculate the relative concentrations of all identified substances based on the peak area of the internal standard. The odor activity value (OAV) was calculated based on the concentration of volatile compounds and the odor threshold (OT) determined in water, and the OT value was obtained through the literature [38,39].

### 2.9. Data Analysis

The experiment adopted a completely random design with three replicates for each group. The SPSS 19.0 software was used for statistical analysis. The significant differences were assessed with the Duncan multiple-range test (*p* ≤ 0.05). Origin 2018 was applied to generate bar charts, heat maps, and radar charts. OPLS and cytoscape software were used for multi-dimensional data analysis in the correlation of antioxidant capacity with phenolic compounds. Principal component analysis (PCA) was performed using the software SIMCA 14.1 to analyze the differences in volatile compounds between the different cultivars.

## 3. Results and Discussion

### 3.1. Soluble Solids and Titratable Acidity

The content of soluble solids and titratable acidity are often used to estimate the maturity and taste of fruits [40]. The soluble solids content and titratable acidity of seven peach cultivars—flat peach (FP), liguang peach (LG), honey peach (HP), yellow peach (YP), yellow nectarine (YN), red nectarine (RN) and shoutao (ST)—were investigated. There were relative significant differences in the titratable acidity and soluble solids content among the tested peaches due to them being different cultivars (*p* ≤ 0.05) (Table 1). The soluble solids content of seven cultivars ranged from 6.96 to 13.47%. Among them, HP had the highest value, followed by FP and YP having the lowest. All the titratable acidity contents were less than 1%, and varied from 0.57 to 0.89% among the seven tested peaches. YN had the least titratable acidity content, while LG had the highest. In the peaches, sweetness was positively correlated with overall taste and was also the main driver of consumer acceptance [41]. In contrast to the other cultivars, HP had highest content of soluble solids and a relatively lower level of acids. These offered HP a better taste and may be the reason for its greater popularity among many consumers.

### 3.2. Total Phenolic and Total Flavonoid

Fruits are good sources of phenols and flavonoids. Furthermore, the content of total phenolic and total flavonoid compounds is associated with antioxidant activity and health-promoting properties [12]. In this work, the total phenolic content and total flavonoid content of the different peach cultivars were determined, and there were relatively significant differences in TPC and TFC among the tested peaches due to them being different cultivars (*p* ≤ 0.05). The total phenolic contents ranged from 33.51 to 62.77 mg GAE/100 g, and the total flavonoid contents varied from 29.05 to 49.37 mg RE/100 g (Table 2). Among the seven various cultivars, ST presented the highest level of TPC and TFC, whereas HP had the lowest value of TPC and YP had the lowest value of TFC. The rich content of phenols and flavonoids in peaches, which serve as natural antioxidants, might be meaningful to dietary research.

### 3.3. Sugars and Organic Acids

The sensory qualities of fruits are significantly influenced by organic acids and sugars [42]. Significant differences among cultivars were found for the contents of sugar and organic acids (*p* ≤ 0.05). Through determination, the sugars in the peach mainly included sucrose, glucose, sorbitol and fructose (Table 3). The total sugar content varied between 69.77 to 118.76 mg/g, with YP having the lowest level and HP having the highest. Seven different peach cultivars ranged in terms of fructose, sorbitol, glucose, and sucrose contents from 6.25 to 14.73 mg/g, 1.19 to 3.72 mg/g, 4.77 to 12.27 mg/g, and 41.98 to 93.68 mg/g, respectively. Fructose, sorbitol, glucose, and sucrose constituted 6.13 to 19.98%, 1.33 to 5.08%, 5.25 to 17.58%, 60.18 to 86.31%, respectively. The difference in fructose content among cultivars was the most significant (*p* ≤ 0.05). Sucrose was the highest of the four sugars (Supporting Information Figure S1) for all seven of the peach cultivars. These results were in line with those that have already been reported for other peach cultivars [43]. Christophe Aubert et al. [6], through an analysis of the composition of four commercial blood-flesh peach cultivars from Europe, revealed that sucrose was the primal sugar detected in all the cultivars. Additionally, in all the tested cultivars, the level of fructose was higher than glucose. By calculating the fructose-to-glucose ratio, we discovered that the ratio ranged from 1.14 to 1.49 and that HP had the highest value. “High-quality” peaches had a larger fructose-to-glucose ratio than “low-quality” peaches, according to a prior study by Byrne et al. [44].

Shown in Table 4, four organic acids were quantified in all seven cultivars, including malic acid, quinic acid succinic acid and citric acid. Malic acid was found as the most abundant organic in peaches and was significantly higher in LG. The content of malic acid ranged between 3.40 to 7.73 mg/g, accounting for 32.41 to 59.14% of the total organic acid. The second-most abundant in the tested peaches was quinic acid, varying from 0.97 to 2.81 mg/g and accounting for 14.75 to 28.97% of the total organic acid. The third-most abundant organic acid was succinic acid, which ranged from 0.43 to 4.13 mg/g and accounted for 5.55 to 37.83% of the total content. The least abundant organic acid was citric acid, which ranged from 0.34 to 1.75 mg/g and accounted for 4.02% to 19.20% of the total content. The findings were in line with other reports [30,45], which found that malic acid was the most abundant acid in all peach cultivars, followed by quinic acid and citric acid. In addition, the total organic acid content ranged from 6.70 to 13.07 mg/g, with LG having the greatest total organic acid and YN having the lowest. According to Christophe Aubert et al. [30], malic acid, accounting for nearly 40% of the total organic acids, was the dominant organic acid found in Western Red nectarines. In this study, the malic acid in RN reached about 65% of the total organic acids.

Previous research showed that malic acid, citric acid and quinic acid were the dominant organic acids in the peach [46,47]. In this study, quinic acid and malic acid were the main organic acids (Supporting Information Figure S2). In addition, this study found that the content of succinic acid was higher than citric acid, which may be due to the unique composition characteristics of organic acids in Xinjiang peaches. Malic acid, quinic acid, and succinic acid were about 4:2:1, accounting for more than 80% of the total organic acids in seven cultivars, which suggested their responsibility for the characteristic acidity of Xinjiang peaches.

It is widely accepted that measuring sugar and acid concentration is an essential basis for predicting the consumer acceptability of fresh fruit [41]. Additionally, the same is true for peaches, as the taste of peach fruit is largely determined by sweetness and sourness. The ratio of sugar to acid is one of the frequently used indexes to measure fruit flavor. The ratio of sugar to acid in peaches ranged from 5.61 to 14.84 (Table 4). LG with the smallest ratio was significantly (about 2.7-fold) lower than HP, which could affect consumer acceptance. In addition, the ratio of sugar to acid in fruits could affect the formation and content of flavor compounds, which might be related to the accumulation mechanism of flavor substances during ripening [48].

### 3.4. Phenolic Compounds

In this work, ten phenolic compounds were determined by means of HPLC analysis of the tested peaches, including *p*-coumaric, procyanidins b, neochlorogenic acid, catechinic, vanillic acid, chlorogenic acid, epicatechin, rutin, quercetin and kaempferol. As shown in Table 5, considerable differences were discovered in the content of total polyphenols, and differences were discovered based on the cultivar (*p* ≤ 0.05). Chlorogenic acid was the major phenolic compound in all the tested cultivars, accounting for 10.43 to 45.50% of all the phenolic compounds, nearly 1.5–10-fold significantly higher compared with the other cultivars. In the seven cultivars, ST had the most abundant content of phenolic compounds, much higher than the other cultivars, and had a significant difference with the others (*p* ≤ 0.05). The second-most abundant phenolic compound in the seven cultivars was neochlorogenic acid, and its concentration varied from 0.17 to 5.68 mg/g, accounting for 8.06 to 24.76% of the total phenolic compounds. This was in line with the results obtained from the study of phenolic compound content in five peach cultivars; chlorogenic acid and neochlorogenic acid were found to be the dominant phenolic compounds in the tested peaches [49].

Compared with the other phenolic compounds, vanillic acid, epicatechin and kaempferol were found in significantly low amounts (Supporting Information Figure S3). In addition, the kaempferol content was minimal; it was only detected in four cultivars of the peaches, namely FP, HP, YN and ST. Much of the benefits of fruit consumption can be attributed to phenolic compounds, which are mainly associated with antioxidant phenolic metabolites [50]. Gil et al. [9] demonstrated that the fruits with higher levels of phenolic compounds had higher antioxidant capacity.

### 3.5. In Vitro Antioxidant Activity

Fruits are a major source of antioxidants, and the antioxidant compounds in plant tissues play a crucial part in their biological effects on humans [51]. In previous studies, in vitro antioxidant capacity has been widely used to assess the biological and nutritional value of fruit. In this study, in order to investigate the in vitro antioxidant potentials of the peach, we used four different methods: ABTS^+^-radical-scavenging activity, DPPH-radical-scavenging activity, FRAP and CUPRAC. The results showed that the ability to scavenge the ABTS^+^ and DPPH free radicals in the seven cultivars of peaches ranged from 155.81 to 312.61 μmol TEs/100 g and 252.68 to 394.55 μmol TEs/100 g, respectively. Particularly, FP had the most effective ABTS^+-^ and DPPH-radical-scavenging activities, whereas RN had the least. Meanwhile, FRAP and CUPRAC in the seven tested peaches varied from 147.81 to 311.79 μmol TEs/100 g and 169.83 to 282.83 μmol TEs/100 g, respectively (Table 6). Similarly, FP was the best at FRAP and CUPRAC, and was 1.16-to-2.1-fold more than the other cultivars (Supporting Information Figure S4). Statistically significant in vitro antioxidant activity values were detected in the seven peach cultivars (*p* ≤ 0.05).

It was suggested that fruits with high antioxidant activity could be vital dietary sources of natural antioxidants to avoiding the diseases caused by oxidative stress [52]. Peaches showed high antioxidant activity in this study, so this may have some implication for research into dietary balance.

### 3.6. Correlation Analysis of Phenolic Compounds and Antioxidant Capacity

**Figure 2:**
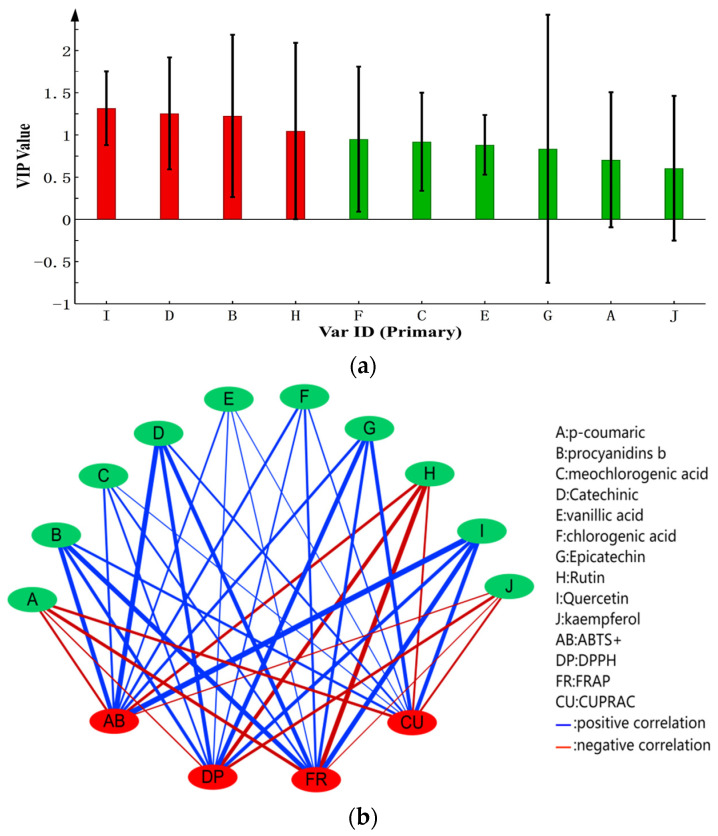
(**a**): The VIP value derived from OPLS analysis characterized the correlation value of the antioxidant capacity. A VIP value greater than 1.0 means significant correlation to the antioxidant capacity. (**b**): Correlations between antioxidant capacities and phenolic compounds in seven peach cultivars. The width of the line is proportional to the strength of the correlation.

Phenols possess antioxidant activity primarily because of their ability to act as reducing agents or free-radical scavengers [53]. OPLS and cytoscape software were used to further clarify the relationship between antioxidant capacity and the phenolic compounds, with the phenolic compounds as independent variables and four indexes of antioxidant activity as dependent variables. The correlation between phenolic compounds and antioxidant activity was established. The results showed that different phenolic compounds had discrepant contributions to antioxidant capacity. The VIP value of quercetin (I), catechinic (D), procyanidins b (B) and rutin (H) were greater than 1, indicating that these phenolic substances were more correlated with antioxidant capacity (Figure 2a), whereas, in the case of the rest of the phenolic substances, the correlation coefficient was lower. The correlation based on a network analysis (|r| > 0.6 with *p* ≤ 0.05) indicated that procyanidins b (B), neochlorogenic acid (C), catechinic (D), vanillic acid (E), chlorogenic acid (F), epicatechin (G) and quercetin (I) were positively correlated with ABTS^+^, DPPH, FRAP, and CUPRAC. However, *p*-coumaric (A), rutin (H) and kaempferol (J) exhibited a negative correlation with all four of the antioxidant abilities (Figure 2b). In addition, different cultivars of peaches may have unique antioxidant abilities.

### 3.7. Volatile Profiles of Seven Peach Cultivars

**Figure 3:**
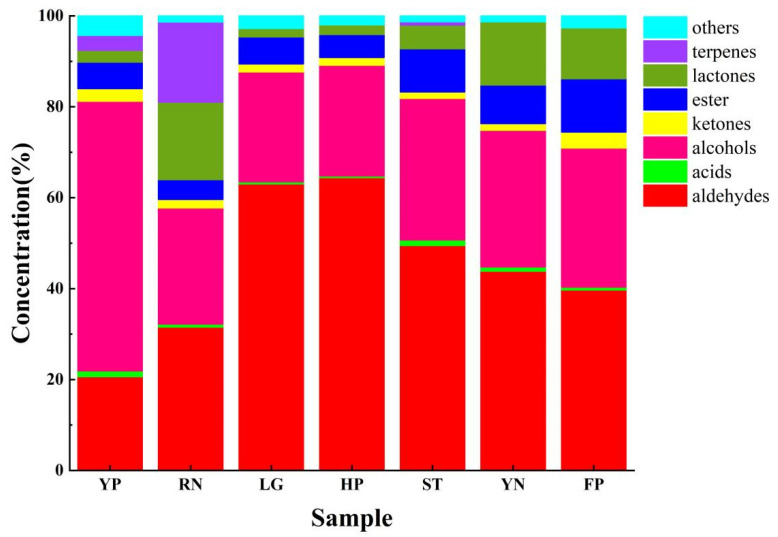
The composition of volatile compounds in seven peach cultivars. Classification of volatile compounds in flat peach (FP), liguang peach (LG), honey peach (HP), yellow peach (YP), yellow nectarine (YN), red nectarine (RN) and shoutao (ST) from shihezi, Xinjiang.

The fruit of separate cultivars, or even the same cultivars, have different volatile components, and the respective compositions and concentrations of volatile compounds endow the fruit with distinctive flavors [16,19]. The volatile organic compounds (VOCs) of peaches were analyzed by the HS-SPME–GC–MS method and the results showed that in sum, 69 aroma compounds were identified and quantified. These compounds can be classified according to chemical structure, including 11 alcohols, 18 aldehydes, 3 acids, 6 ketones, 15 esters, 8 lactones, 4 terpenes, and 4 other compounds. A sum of 37 volatiles were present in YP, 46 volatiles in RN, 41 volatiles in LG, 31 volatiles in HP, 38 volatiles in ST, 44 volatiles in YN and 37 volatiles in FP, respectively (Supporting Information Table S1). Alcohols and aldehydes had the most abundant relative contents in all the peaches, representing 20.64 to 64.43% and 24.20 to 59.30% of the total VOC content, respectively. Furthermore, the concentrations of volatile compounds differed largely among the seven cultivars (Figure 3).

#### 3.7.1. Principal Component Analysis

**Figure 4:**
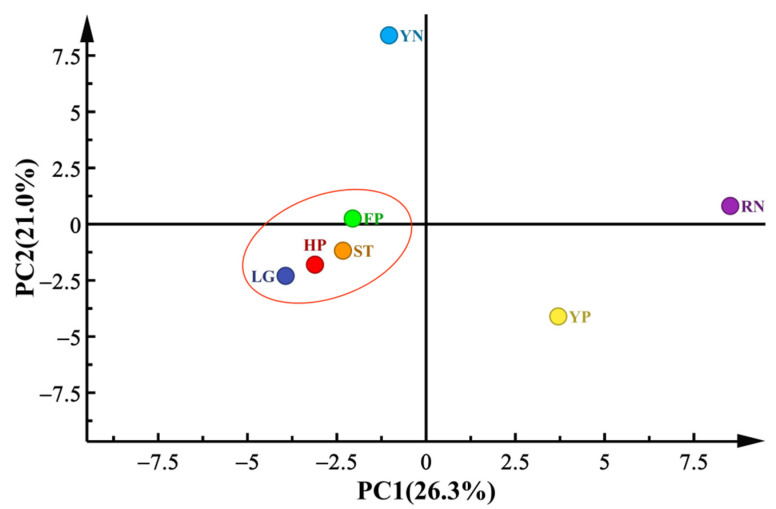
PCA analysis of aroma compounds. Differences in the flavor compounds in flat peach (FP), liguang peach (LG), honey peach (HP), yellow peach (YP), yellow nectarine (YN), red nectarine (RN) and shoutao (ST) from shihezi, Xinjiang.

Principal component analysis (PCA) was used to visualize the difference in aroma compounds among the seven peach cultivars. The percentage of the cumulative contribution of variance of the two PCs was 47.31%; apart, PC1 presented 26.3% and PC2 presented 21.0% (Figure 4). The cultivar of peach seemed to be the dominant factor in the differentiation. Depending on the degree of the dispersion of the cultivars, the seven tested peach cultivars could be divided into four categories. In the score plot, FP, LG, HP and ST were grouped into cluster 1 because they were relatively close together and seemed to hold similar aroma characteristics. YP was grouped into cluster 2 because it contained more alcohols than the other cultivars, such as hexanol and hotrienol, which endowed more of a green odor. YN was in cluster 3 due to the higher concentration of lactones that were reminiscent of a creamy and peach-like odor. Finally, RN was clustered into group 4 with a peculiar abundance of terpenes, such as styrene and linalool, which endowed RN with more of a sweet and floral scent (Supporting Information Figure S5). The results showed that more than half of the tested cultivars were clustered together, suggesting that different peach cultivars possessed similar aroma characteristics.

#### 3.7.2. Aroma Compound Composition and Content

The composition and content of the aroma components in the seven peaches were different among cultivars. The total volatile contents were greater than 1100 μg/kg for all of the tested peaches except the HP cultivar. It is very interesting to note that LG had the highest volatile content (1413.29 μg/kg) among all the investigated peach cultivars. Peach aroma compounds contained a large number of C6 compounds. In this study, 10 kinds of C6 compounds were detected, namely hexanal, hexanol, 3-hexene-1-ol, 4-pentenal-2-methyl-, 2-hexen-1-ol, 2-hexenal, 5-methyl furfural, 5-hydroxymethylfurfural, γ-caprolactone and 2-ethylfuran, among which 2-hexenal was the most abundant. The highest C6 compounds were detected in HP, reaching 909.15 μg/kg and accounting for 65.12% of the total aroma. Several lines of evidence suggested that C6 compounds contributed to the green fragrance in the peach, and the content decreased with peach ripening and prolonged storage [30,54].

Esters and lactones contribute to the fruity and floral odor and are essential aroma compounds in fruit. The lactone compounds detected in the seven cultivars were γ-Caprolactone, γ-Octalactone, δ-Octalactone, γ-Nonalactone, γ-Decalactone, γ-Heptalactone, δ-Decalactone and γ-Dodecalacton—a total of 8 compounds—making up 1.85 to 17.18% of the total aroma (Supporting Information Table S2). Especially HP had the greatest abundance of lactones (236.83 μg/kg). Among them, there were three kinds of lactones commonly detected, namely γ-Caprolactone, γ-Decalactone and δ-Decalactone, and δ-Decalactone was the most abundant lactone compound in all the cultivars. This was consistent with previous studies [55]. Additionally, a total of six esters were commonly found in the tested peaches, namely hexyl acetate, 3-hexen-1-ol acetate, cis-3-hexenyl acetate, ethyl caprylate, ethyl benzoate and phenethyl acetate. A higher phenethyl acetate content (44.42 μg/kg) was observed in FP compared to the other cultivars. Further analysis showed that in addition to the common volatiles, methyl octylate was exclusively detected in HP, p-tolyl acetate was exclusively detected in LG, 2-phenylethyl octanoate was specifically detected in YN and ethyl phenylacetate was specifically detected in FP.

Aldehydes and alcohols, as the dominant aroma components in the peach, provide the overall green odor. Up to 11 kinds of alcohols and 18 kinds of aldehydes were detected in the seven cultivars. The categories and concentrations of aldehydes and alcohols varied greatly among the different cultivars. Among them, seven common volatiles were detected in all the cultivars, namely hexanol, n-hexanal, 2-hexenal, nonanal, decanal, benzaldehyde and 2,6-nonadienal. Especially, benzaldehyde had the most abundant concentration, varying from 335.93 to 963.57 μg/kg, which was characterized by a “green” and “honey” odor. Terpenes contribute to the fresh floral flavor of the peach and were only found in the YP, RN and ST cultivars in this study. Additionally, four terpenes were detected in all the cultivars, namely β-myrcene, limonene, styrene and linalool, accounting for 0.75 to 17.74% of the total aroma compounds. Differently, linalool was only detected in the RN sample in this work, but it has been reported to be an important aroma compound in previous studies [21,56].

In addition, three acids, six ketones and four other compounds were detected. These substances were present, but their significance was relatively minimal, accounting for 0.36 to 1.28%, 1.42 to 3.51% and 1.32 to 4.30% of total aroma compounds, respectively. Among them, there were four common aroma compounds found in the seven cultivars, called acetic acid, dihydro-β-ionone, geranylaceton and 6-pentylpyran-2-one.

A total of 20 common aroma compounds were found in the seven peach cultivars (Table 7). In order to construct the characteristic aroma of the peach, these 20 common volatile compounds were selected for further analysis.

#### 3.7.3. Construction of Peach Aroma Heat Map

**Figure 5:**
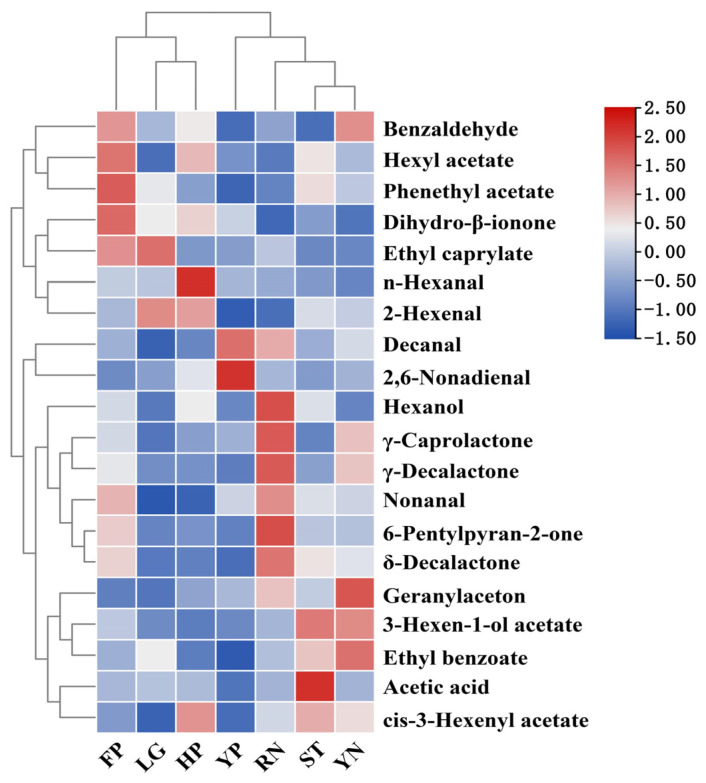
The heat map of the main aroma compounds in seven peach cultivars. The OAVs of the main aroma compounds in flat peach (FP), liguang peach (LG), honey peach (HP), yellow peach (YP), yellow nectarine (YN), red nectarine (RN) and shoutao (ST) from shihezi, Xinjiang.

The main aroma components in peaches of different cultivars were investigated by cluster heat-map analysis. The 20 common volatile compounds were analyzed by constructing a heat map. According to the results of the sample cluster, the seven peach cultivars were clustered into two categories based on the difference in the concentration of volatile compounds (Figure 5). There were different characteristic odors in each cluster. Among them, the ST, YN, YP and RN had the prominent sweet, typical green and nutty flavors and FP, LG and HP had the characteristic floral, citrus and fatty aromas. Among all the aroma compounds, lactones have the distinctive peach odor, which is one of the crucial aroma characteristics that distinguish peach from other fruits.

#### 3.7.4. Identification of Peach Aroma Fingerprint

A total of twenty common aroma compounds were identified, but not all of them contributed significantly to the aroma of the peaches; only those with an OAV above 1 were considered important to the aroma [16]. The odor activity value (OAV) is the ratio of the volatile compound concentration to the odor threshold (OT), and is used to distinguish which compound contributes more to fruit aroma. Only two of the esters found to be volatile in the seven cultivars had OAV values above 1—hexyl acetate and cis-3-hexenyl acetate—indicating that they played a significant role in the fruity and floral notes of the peach. Although other often-found esters had distinctive smells as well, their contributions to the peach aroma were minimal because of their low concentrations and high odor sensitivities. For the common lactone volatiles, γ-decalactone was reminiscent of a fruity, pleasant and peach-like odor and had a relatively low odor threshold (0.7 µg/kg), so it had OAVs ranging from 8.77 to 252.17 (Table 7), making it a crucial contributor to the peach aroma among the lactones. For the other lactones, δ-decalactone is characterized by a coconut odor and flavor and γ-caprolactone is characterized by a creamy and nutty caramel odor, but their OAVs in the seven cultivars were below 1. Especially, according to reports, γ-decalactone is a key component in the creation of the distinctive peach-like scent [56].

There were a total of seven kinds of aldehydes and alcohols commonly detected in tested peach, and five of them had OAVs greater than 1. The aldehyde and alcohol compounds with OAVs above 1 included n-hexanal, 2-hexenal, nonanal, decanal and benzaldehyde, which implied that these compounds gave the peach a stronger green, fruity, sweet, fatty, and citrus-like odor than the others. For other common volatile compounds, only 6-pentylpyran-2-one had an OAV greater than 1, which was characterized by a mushroom and cheese odor.

**Figure 6:**
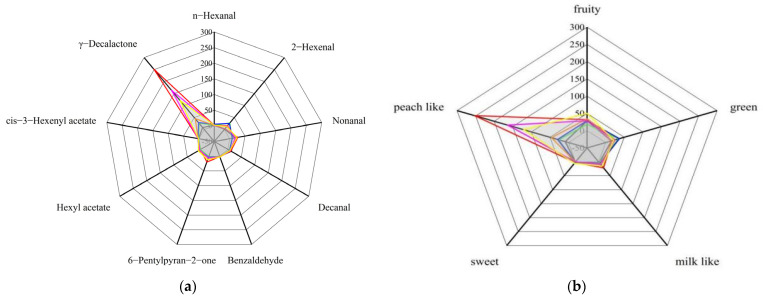
(**a**): The flavor analysis of seven peach cultivars. Nine important aroma compounds of flat peach (yellow line), liguang peach (green line), honey peach (blue line), yellow peach (black line), yellow nectarine (purple line), red nectarine (red line) and shoutao (orange line). (**b**): Flavor analysis of the main volatile compounds in seven peach cultivars. The flavor analysis of flat peach (yellow line), liguang peach (green line), honey peach (blue line), yellow peach (black line), yellow nectarine (purple line), red nectarine (red line) and shoutao (orange line).

To sum up, the OAV values of nine compounds were above 1, and these key aroma compounds were used to construct the aroma fingerprint of peaches. The aroma fingerprint of the peach (OAVs > 1) was composed of hexyl acetate, cis-3-hexenyl acetate, γ-decalactone, n-hexanal, 2-hexenal, nonanal, decanal benzaldehyde and 6-pentylpyran-2-one (Supporting Information Figure S6). There were still significant differences in the nine key aroma compounds among the seven cultivars of peaches. The aroma characteristics of the seven cultivars of peaches seemed to be unique (Figure 6). The shaded region is the fiducial interval of the important aroma compounds in the seven peaches. The aroma of different cultivars of peaches had similar characteristics. Each aroma compound was not working alone, but rather they combined with each other to endow the peach with a unique aroma characteristic [21]. According to the flavor characteristics of volatile compounds, the aroma presented by the main aroma compounds can be divided into fruity, green, milk-like, sweet and peach-like. As shown in Figure 6, through the higher content and lower threshold of lactones, the aroma characteristics of peaches in shihizi, Xinjiang mainly tended to be peach-like.

## 4. Conclusions

In this study, data were obtained through investigations of the physicochemical, functional and aroma characteristics of different peach cultivars in Xinjiang. The results showed that fructose and sucrose, which accounted more than 70% of the total sugars, and malic acid, quinic acid and succinic acid, which accounted for over 80% of the total organic acids, were the dominant contributors to sweetness and acidity in the seven peaches. Moreover, by using the method described in this study, most of the phenolic compounds were positively correlated with antioxidant activity. Meanwhile, the aroma fingerprint of the peach was constructed, and these aroma components can be used as specific markers to distinguish the aroma of the peach from other fruits.

By analyzing the properties of different cultivars of peaches, a theoretical basis can be established for the development of peach-flavored foods in practical production. However, the study of the peach aroma characteristic in this work was only a prediction based on multivariate data and an OAV analysis. In future research, e-nose and e-tongue equipment, as well as aroma recombination technology can be used to further identify and qualify the flavor compounds in peaches, and provide a theoretical basis for the development of the aroma components in peaches.

## Figures and Tables

**Table 1:** The soluble solids content and titratable acidity of seven peach cultivars.

Cultivar	SSC ^1^ (%)	TA ^2^ (%)
FP	12.20 ± 0.05 ^b^	0.81 ± 0.02 ^bc^
LG	7.49 ± 0.02 ^e^	0.89 ± 0.04 ^a^
HP	13.47 ± 0.02 ^a^	0.79 ± 0.01 ^c^
YP	6.96 ± 0.07 ^f^	0.82 ± 0.01 ^b^
YN	7.55 ± 0.03 ^e^	0.57 ± 0.03 ^e^
RN	8.84 ± 0.03 ^d^	0.67 ± 0.01 ^d^
ST	9.21 ± 0.04 ^c^	0.69 ± 0.02 ^d^

^1^ SSC, soluble solids content. ^2^ TA, titratable acidity (TA). Values are presented as mean ± standard deviation (*n* = 3). The different superscripts in the column indicate significant differences (Duncan, *p* ≤ 0.05) for different peach cultivars.

**Table 2:** The total phenolic and total flavonoid content of seven peach cultivars.

Cultivar	TPC ^1^ (mg GAE/100 g)	TFC ^2^ (mg RE/100 g)
FP	58.92 ± 1.29 ^b^	49.30 ± 0.02 ^a^
LG	59.37 ± 0.40 ^b^	37.28 ± 0.03 ^b^
HP	33.51 ± 2.19 ^e^	29.16 ± 0.05 ^d^
YP	36.20 ± 0.80 ^c^	29.05 ± 0.03 ^e^
YN	34.58 ± 0.39 ^d^	34.53 ± 0.07 ^c^
RN	35.26 ± 1.42 ^cd^	34.58 ± 0.05 ^c^
ST	62.77 ± 0.95 ^a^	49.37 ± 0.01 ^a^

^1^ TPC, total phenolic content. ^2^ TFC, total flavonoid content. Values are presented as mean ± standard deviation (*n* = 3). The different superscripts in the column indicate significant differences (Duncan, *p* ≤ 0.05) for different peach cultivars.

**Table 3:** Sugars (mg/g) in flesh of the seven peach cultivars.

Cultivar	Fructose	Sorbitol	Glucose	Sucrose	Total
FP	6.58 ± 0.49 ^e^	2.25 ± 0.12 ^c^	5.64 ± 0.32 ^d^	90.88 ± 1.15 ^b^	105.35 ± 1.93 ^b^
LG	9.82 ± 0.43 ^d^	3.72 ± 0.16 ^a^	7.52 ± 0.49 ^c^	52.24 ± 1.07 ^f^	73.30 ± 1.08 ^f^
HP	12.01 ± 0.30 ^c^	3.17 ± 0.28 ^b^	9.90 ± 0.31 ^b^	93.68 ± 1.33 ^a^	118.76 ± 0.96 ^a^
YP	13.94 ± 0.32 ^b^	1.57 ± 0.13 ^d^	12.27 ± 0.38 ^a^	41.98 ± 0.51 ^g^	69.77 ± 0.40 ^g^
YN	9.75 ± 0.33 ^d^	1.39 ± 0.07 ^de^	7.92 ± 0.54 ^c^	68.12 ± 1.40 ^d^	87.18 ± 0.47 ^d^
RN	6.25 ± 0.48 ^e^	1.19 ± 0.10 ^e^	4.77 ± 0.31 ^e^	76.95 ± 1.35 ^c^	89.16 ± 0.67 ^c^
ST	14.73 ± 0.37 ^a^	1.36 ± 0.12 ^de^	9.83 ± 0.85 ^b^	54.84 ± 0.72 ^e^	80.76 ± 0.62 ^e^

Note: Values are presented as mean ± standard deviation (*n* = 3). The different superscripts in the column indicate significant differences (Duncan, *p* ≤ 0.05) for different peach cultivars.

**Table 4:** Organic acids (mg/g) in flesh of the seven peach cultivars.

Cultivar	Quinic Acid	Malic Acid	Citric Acid	Succinic Acid	Total	Sugar/Acid
FP	2.81 ± 0.21 ^a^	3.54 ± 0.08 ^de^	0.44 ± 0.01 ^f^	4.13 ± 0.13 ^a^	10.93 ± 0.23 ^b^	9.64 ± 0.49 ^c^
LG	1.93 ± 0.12 ^d^	7.73 ± 0.28 ^a^	1.51 ± 0.10 ^b^	1.90 ± 0.15 ^b^	13.07 ± 0.17 ^a^	5.61 ± 0.62 ^e^
HP	2.00 ± 0.12 ^cd^	3.79 ± 0.14 ^d^	0.91 ± 0.03 ^c^	1.31 ± 0.05 ^d^	8.01 ± 0.16 ^d^	14.84 ± 0.25 ^a^
YP	2.34 ± 0.13 ^b^	3.53 ± 0.13 ^de^	1.75 ± 0.13 ^a^	1.48 ± 0.05 ^c^	9.10 ± 0.18 ^c^	7.66 ± 0.65 ^d^
YN	0.97 ± 0.09 ^f^	3.40 ± 0.12 ^e^	0.73 ± 0.02 ^d^	1.58 ± 0.05 ^c^	6.70 ± 0.19 ^f^	13.02 ± 0.72 ^b^
RN	1.55 ± 0.11 ^e^	4.93 ± 0.18 ^b^	0.34 ± 0.01 ^g^	0.64 ± 0.03 ^e^	7.47 ± 0.25 ^e^	11.93 ± 0.31 ^b^
ST	2.24 ± 0.13 ^bc^	4.52 ± 0.16 ^c^	0.55 ± 0.02 ^e^	0.43 ± 0.02 ^f^	7.74 ± 0.12 ^de^	10.43 ± 0.09 ^c^

Note: Values are presented as mean ± standard deviation (*n* = 3). The different superscripts in the column indicate significant differences (Duncan, *p* ≤ 0.05) for different peach cultivars.

**Table 5:** Phenolic compounds (mg/g) of seven peach cultivars.

Cultivar	FP	LG	HP	YP	YN	RN	ST
*p*-coumaric	0.63 ± 0.01 ^b^	1.22 ± 0.10 ^a^	0.46 ± 0.01 ^c^	0.30 ± 0.02 ^d^	0.27 ± 0.01 ^de^	0.20 ± 0.01 ^e^	0.19 ± 0.01 ^e^
procyanidins b	1.96 ± 0.13 ^b^	0.94 ± 0.12 ^c^	1.03 ± 0.09 ^c^	1.98 ± 0.06 ^b^	0.45 ± 0.04 ^d^	0.60 ± 0.08 ^d^	6.65 ± 0.37 ^a^
neochlorogenic acid	0.76 ± 0.04 ^d^	3.60 ± 0.18 ^b^	0.62 ± 0.01 ^d^	1.70 ± 0.02 ^c^	0.17 ± 0.01 ^e^	1.54 ± 0.04 ^c^	5.68 ± 0.37 ^a^
catechinic	1.81 ± 0.05 ^b^	1.00 ± 0.03 ^c^	1.83 ± 0.05 ^b^	1.84 ± 0.12 ^b^	0.39 ± 0.01 ^d^	0.38 ± 0.01 ^d^	4.62 ± 0.14 ^a^
vanillic acid	0.08 ± 0.01 ^c^	0.14 ± 0.01 ^a^	0.11 ± 0.02 ^b^	0.05 ± 0.01 ^de^	0.04 ± 0.01 ^e^	0.06 ± 0.01 ^d^	0.16 ± 0.01 ^a^
chlorogenic acid	3.25 ± 0.19 ^c^	6.55 ± 0.20 ^b^	0.98 ± 0.08 ^d^	0.87 ± 0.06 ^d^	0.22 ± 0.01 ^e^	2.83 ± 0.30 ^c^	10.22 ± 0.59 ^a^
epicatechin	0.44 ± 0.01 ^a^	0.29 ± 0.01 ^b^	0.17 ± 0.01 ^e^	0.27 ± 0.01 ^c^	0.06 ± 0.01 ^f^	n.d	0.24 ± 0.01 ^d^
rutin	0.34 ± 0.01 ^d^	0.86 ± 0.06 ^a^	0.38 ± 0.03 ^c^	0.08 ± 0.01 ^f^	0.29 ± 0.01 ^e^	0.54 ± 0.01 ^b^	0.12 ± 0.01 ^f^
quercetin	0.22 ± 0.01 ^b^	0.08 ± 0.01 ^d^	0.15 ± 0.01 ^c^	0.14 ± 0.01 ^c^	0.14 ± 0.01 ^c^	0.07 ± 0.01 ^d^	0.35 ± 0.02 ^a^
kaempferol	0.03 ± 0.01 ^c^	n.d	0.02 ± 0.01 ^d^	n.d	0.08 ± 0.01 ^a^	n.d	0.05 ± 0.01 ^b^
total	9.51 ± 0.18 ^c^	14.68 ± 0.09 ^b^	5.75 ± 0.28 ^f^	7.22 ± 0.12 ^d^	2.11 ± 0.05 ^g^	6.22 ± 0.21 ^e^	28.28 ± 0.47 ^a^

Note: Values are presented as mean ± standard deviation (*n* = 3). The different superscripts in the column indicate significant differences (Duncan, *p* ≤ 0.05) for different peach cultivars.

**Table 6:** Antioxidant capacities (μmol TEs/100 g) of the seven peach cultivars.

Cultivar	Radical-Scavenging Capacity		Reducing Capacity	
	ABTS^+^	DPPH	FRAP	CUPRAC
FP	312.61 ± 0.23 ^a^	394.55 ± 0.13 ^a^	311.79 ± 0.30 ^a^	282.83 ± 0.21 ^a^
LG	197.56 ± 0.01 ^f^	293.52 ± 0.34 ^e^	149.24 ± 0.18 ^f^	169.83 ± 0.10 ^f^
HP	203.21 ± 0.24 ^e^	296.42 ± 0.19 ^d^	153.07 ± 0.08 ^e^	185.03 ± 0.07 ^e^
YP	236.91 ± 0.03 ^c^	390.84 ± 0.13 ^b^	243.57 ± 0.21 ^c^	204.84 ± 0.04 ^c^
YN	227.46 ± 0.14 ^d^	280.95 ± 0.07 ^f^	189.93 ± 0.19 ^d^	201.49 ± 0.13 ^d^
RN	155.81 ± 0.18 ^g^	252.68 ± 0.15 ^g^	147.81 ± 0.11 ^g^	184.99 ± 0.27 ^e^
ST	311.99 ± 0.04 ^b^	384.53 ± 0.20 ^c^	306.86 ± 0.19 ^b^	243.16 ± 0.17 ^b^

Note: Values are presented as mean ± standard deviation (*n* = 3). The different superscripts in the column indicate significant differences (Duncan, *p* ≤ 0.05) for different peach cultivars.

**Table 7:** OAVs of main volatile compounds found in flesh of seven peaches.

Volatile Compounds	RI	FP	LG	HP	YP	YN	RN	ST	OT ^1^ (μg/kg)	Flavor Description
**Esters and lactones**										
Hexyl acetate	1276	9.53	1.22	7.51	2.53	1.65	4.03	6.27	2	Sweet, fruity
3-Hexen-1-ol acetate	1320	1.12	0.31	0.13	0.28	0.87	2.64	2.83	7.8	Banana
cis-3-Hexenyl acetate	1337	1.71	1.13	3.31	1.24	2.33	2.72	3.11	8	Fruity, green
Ethyl caprylate	1436	5.27	6.02	0.80	0.96	2.11	0.31	0.36	5	Banana, pear
Ethyl benzoate	1658	0.07	0.08	0.06	0.05	0.07	0.11	0.09	60	Floral, sweet
Phenethyl acetate	1808	0.01	<0.01	<0.01	<0.01	<0.01	<0.01	<0.01	3000	Rose, sweet
γ-Caprolactone	1709	0.04	<0.01	0.02	0.02	0.07	0.05	0.01	260	Milk-like, nutty
γ-Decalactone	2109	120.36	26.15	29.15	8.77	252.17	163.58	45.21	0.7	Peach-like
δ-Decalactone	2193	0.68	0.09	0.12	0.05	0.99	0.54	0.63	31	Cream, peach-like
**Alcohols and aldehydes**										
Hexanol	1341	0.01	<0.01	0.01	<0.01	0.02	<0.01	0.01	500	Fruity, fatty
n-Hexanal	1093	2.34	2.24	5.14	1.99	1.83	1.27	1.57	21	Green, herb
2-Hexenal	1213	11.81	26.82	25.18	1.22	3.26	14.10	15.81	30	Green
Nonanal	1396	23.92	7.75	8.71	17.95	263.90	17.93	18.91	1	Orange, rose, fat
Decanal	1506	7.83	5.57	6.64	12.53	11.15	9.04	7.76	1	Orange
Benzaldehyde	1546	2.96	1.76	2.32	1.05	1.57	3.01	1.07	320	Nutty
2,6-Nonadienal	1715	0.75	0.16	0.09	0.29	0.08	0.15	0.04	17.28	Green, cucumber
**Others**										
Acetic acid	1492	<0.01	<0.01	<0.01	<0.01	<0.01	<0.01	<0.01	22,000	Sour
Dihydro-β-ionone	1825	1.38	0.12	1.74	2.00	0.74	0.27	2.98	7	Floral, fruity, wood
Geranylaceton	1840	0.04	0.03	0.05	0.06	0.09	0.13	0.07	60	Fruity
6-Pentylpyran-2-one	2175	12.41	2.17	3.37	1.97	19.99	6.89	7.23	0.9	Cheese, milk

^1^ OT, Odor threshold.

## Data Availability

Data are contained within the article.

## Supplementary Materials

The following supporting information can be downloaded at: https://www.mdpi.com/article/10.3390/foods11192944/s1. Table S1: Concentration (μg/kg) of aroma compounds found in seven peach cultivars; Table S2: Concentration (%) of aroma compounds in seven peach cultivars; Figure S1: The relative concentration of sugar in seven peach cultivars; Figure S2: The relative concentration of organic acid in seven peach cultivars; Figure S3: The relative concentration of phenolic compound in seven peach cultivars; Figure S4: The antioxidant capacities in seven peach cultivars; Figure S5: The loading diagram of aroma compounds in seven peach cultivars; Figure S6: The ion chromatograms of the seven peach cultivars.
