# Supplementary material for: Physicochemical Characteristics, Antioxidant Activities, and Aroma Compound Analysis of Seven Peach Cultivars (Prunus persica L. Batsch) in Shihezi, Xinjiang
Source: Foods. 2022 Sep 20;11(19):2944. doi: 10.3390/foods11192944 (PMC9563965; doi:10.3390/foods11192944)
Supplement: Supplementary file 1 [file foods-11-02944-s001.zip › foods-1916597-supplementary.pdf]

foods

Supplementary Materials

Physicochemical Characteristics, Antioxidant Activities, and Aroma Compound Analysis of Seven Peach Cultivars (*Prunus persica* L. *Batsch*) in Shihezi, Xinjiang

Huimin Wu, Youyou Xu, Huan Wang, Yuanyuan Miao, Chunyan Li, Ruirui Zhao, Xuewei Shi and Bin Wang *

Food College, Shihezi University, Shihezi 832000, China
* Correspondence: binwang0228@shzu.edu.cn; Tel.: +86-0993-2058-093

Contents

Supplementary Tables 2
Table S1. Concentration (μg/kg) of aroma compounds found in seven peach cultivars........ 2
Table S2. Concentration (%) of aroma compounds in seven peach cultivars. 6
Supplementary Figures........ 6
Figure S1. The relative concentration of sugar in seven peach cultivars. 6
Figure S2. The relative concentration of organic acid in seven peach cultivars. 7
Figure S3. The relative concentration of phenolic compounds in seven peach cultivars. 7
Figure S4. The antioxidant capacities in seven peach cultivars. 8
Figure S5. The loading diagram of aroma compounds in seven peach cultivars. 8
Figure S6. The ion chromatograms of the seven peach cultivars. 9

Supplementary Tables

Table S1. Concentration (μg/kg) of aroma compounds found in seven peach cultivars.

	RI[1]	YP	RN	LG	HP	ST	YN	FP	OT[2](μg /kg)	OAV	Odors
**Alcohols**											
Hexanol	1341	1.92±0.07d	9.82±0.46a	1.46±0.08e	5.44±0.05b	4.98±0.14c	1.80±0.08de	4.73±0.36c	500	<0.1	Fruity, green
3-Hexene-1-ol	1394	n.d	n.d	n.d	n.d	0.72±0.02a	n.d	n.d	910	<0.1	Resinous, floral, born green
2-Hexen-1-ol	1398	n.d	2.02±0.09d	5.03±0.26b	1.33±0.01e	11.41±0.32a	3.08±0.14c	3.02±0.23c	400	<0.1	n.f
1-Nonanol	1652	n.d	n.d	n.d	n.d	n.d	0.80±0.04a	n.d	50	<0.1	Rose, citrus, blossom
1-Octen-3-ol	1454	0.48±0.02c	1.90±0.09a	n.d	n.d	0.32±0.01d	0.60±0.03b	n.d	1	0.1-1	Mushroom, lavender, rose
2-Ethylhexanol	1472	n.d	2.57±0.12a	n.d	n.d	n.d	n.d	n.d	270000	<0.1	Green
1-Octanol	1558	1.94±0.07c	n.d	1.21±0.06d	1.88±0.02c	n.d	3.94±0.18a	2.32±0.18b	4.05	0.1-1	Citrus, oil
Hotrienol	1616	n.d	5.54±0.26a	2.64±0.14b	n.d	n.d	0.19±0.01de	1.95±0.15c	n.f	—	Green, sweet, wood
2-Octen-1-ol	1603	0.17±0.01c	1.95±0.09a	0.38±0.02b	n.d	n.d	n.d	n.d	40	<0.1	n.f
2-Nonen-1-ol	1722	n.d	0.89±0.04a	n.d	n.d	n.d	0.30±0.01b	n.d	n.f	—	Fatty, violet
Dihydro-β-ionol	1976	0.57±0.02c	n.d	n.d	1.22±0.01a	1.10±0.03b	n.d	n.d	n.f	—	Wood, floral
**Acids**											
Acetic acid	1492	2.08±0.08d	4.78±0.23c	5.40±0.28b	5.09±0.05bc	13.68±0.38a	4.77±0.22c	4.97±0.38bc	22000	<0.1	Sour
Isovaleric acid	1660	5.21±0.19a	2.75±0.13b	n.d	n.d	n.d	n.d	n.d	33	0.1-1	Fruity, citrus
Creamy lactone	2226	n.d	1.84±0.09b	1.01±0.05c	n.d	n.d	5.82±0.27a	1.75±0.13b	n.f	—	Jasmine, fruity
**Aldehydes**											
Hexanal	1093	41.79±4.35c	38.53±0.09d	47.08±2.47bc	107.95±0.92a	33.00±0.92e	26.59±1.22f	49.24±3.78b	21	>1	Green, herb
4-Pentenal, 2-methyl-	1141	n.d	n.d	n.d	10.07±0.08a	n.d	n.d	n.d	n.f	—	n.f
2-Hexenal	1213	36.52±0.49g	97.86±4.63f	804.63±42.30a	755.48±6.44b	474.27±13.23c	423.13±19.48d	354.30±27.19e	30	>1	Green
Octanal	1287	3.09±0.11a	2.13±0.10c	n.d	0.54±0.01e	0.90±0.03d	2.65±0.12b	n.d	0.7	>1	Rose, flavedo
Nonanal	1396	17.95±0.65b	263.90±12.49a	7.75±0.41c	8.71±0.07c	18.91±0.53b	17.93±0.82b	23.92±1.83b	1	>1	Orange, rose, fatty
2-Octenal	1428	11.61±0.42a	8.88±0.42b	n.d	n.d	2.32±0.06c	n.d	n.d	3	>1	Fatty, chicken, cucumber

2,4-Heptadienal	1494	9.69±0.35a	7.94±0.37b	5.73±0.30d	4.06±0.03e	6.64±0.19c	3.21±0.15f	0.95±0.07g	56	0.1-1	Green, fatty, citrus
Decanal	1506	12.53±0.45a	11.15±0.53b	5.57±0.29f	6.64±0.06e	7.76±0.22d	9.04±0.42c	7.83±0.60d	1	>1	Citrus
Benzaldehyde	1546	335.93±7.39e	502.47±23.79d	564.16±29.66c	744.29±6.35b	342.27±13.80e	963.57±44.36a	946.32±32.43a	24	>1	Bitter almond
2-Nonenal	1548	22.96±0.83a	21.53±1.02b	n.d	n.d	5.24±0.15d	8.43±0.39c	n.d	0.4	>1	Cardboard taste
5-Methyl furfural	1610	n.d	0.51±0.20a	n.d	n.d	n.d	n.d	n.d	n.f	—	Spicy, sweet, caramel
2,6-Nonadienal	1715	12.93±0.46a	2.80±0.13c	1.65±0.09d	5.05±0.04b	1.43±0.04d	2.61±0.12c	0.66±0.05e	17.28	0.1-1	Green, cucumber
β-Cyclocitral	1610	n.d	3.06±0.14a	n.d	n.d	n.d	n.d	n.d	n.f	—	Fruity, green
Phenylacetaldehyde	1722	2.19±0.08c	2.62±0.12b	11.17±0.59a	n.d	1.23±0.03d	0.65±0.03e	n.d	4	0.1-1	Hyacinth, fragrance
2,4-Decadienal	1766	n.d	n.d	n.d	0.98±0.01a	n.d	0.51±0.02b	n.d	0.07	>1	Citrus, fatty
2,5-Dimethylbenzaldehyde	1775	0.49±0.02e	0.98±0.05d	1.48±0.08c	n.d	2.28±0.06a	1.46±0.07c	2.10±0.16b	40	<0.1	n.f
Pentanal	988	n.d	10.52±0.50a	4.72±0.25b	n.d	n.d	n.d	4.77±0.37b	20	0.1-1	Fruity, nutty
5-Hydroxymethylfurfural	2528	n.d	n.d	0.60±0.03a	n.d	n.d	n.d	n.d	282	<0.1	Chamomile fragrance
**Ketones**											
3-Octanone	1251	0.68±0.02a	n.d	n.d	n.d	n.d	n.d	n.d	21	<0.1	Fruity
Dihydro-β-ionone	1825	9.68±0.35d	0.86±0.04f	12.20±0.64c	14.01±0.12b	5.18±0.15e	1.90±0.09f	20.88±1.60a	0.7	>1	Sweet, floral
Geranylaceton	1840	3.60±0.13d	5.82±0.28b	1.91±0.09f	3.08±0.02e	4.11±0.11c	7.99±0.37a	2.15±0.16f	60	<0.1	Sweet, rose
Dehydrodihydroionone	1865	n.d	n.d	0.48±0.03a	n.d	n.d	n.d	n.d	3.6	0.1-1	Floral, fruity
β-ionone	1954	n.d	1.01±0.05d	8.12±0.43a	3.75±0.03c	n.d	0.64±0.03e	4.99±0.38b	3.5	0.1-1	Floral, violet, woody
6-Pentylpyran-2-one	2175	1.77±0.06e	17.99±0.85a	1.96±0.10e	3.03±0.03d	6.51±0.18c	6.20±0.29c	11.17±0.86b	0.9	>1	Mushroom, cheese
**Esters**											
2-Phenylethyl caproate	2160	n.d	n.d	6.17±0.32c	n.d	7.64±0.21b	12.91±0.59a	n.d	250	<0.1	Fruity, pineapple

Hexyl acetate	1276	5.06±0.18e	3.30±0.16f	2.44±0.13f	15.02±0.13b	12.53±0.35c	8.06±0.37d	19.05±1.46a	2	>1	Fruity, sweet
cis-3-Hexenyl acetate	1320	2.21±0.08e	6.76±0.32d	2.38±0.13e	0.99±0.01f	22.07±0.62a	20.60±0.95b	8.71±0.67c	7.8	0.1-1	Green, grassy
Hex-3-enyl acetate	1337	9.95±0.36f	18.67±0.88d	9.06±0.48f	26.50±0.23a	24.88±0.69b	21.76±100c	13.69±1.05e	8	>1	Fruity
Methyl octylate	1398	n.d	n.d	n.d	5.15±0.04a	n.d	n.d	n.d	n.f	—	Fruity, citrus
Ethyl caprylate	1436	4.78±0.17d	10.57±0.50c	30.09±1.58a	4.00±0.03d	1.79±0.05e	1.57±0.07e	26.33±2.02b	5	0.1-1	Fruity, pineapple, apple
Heptylacetat	1377	n.d	n.d	n.d	n.d	n.d	0.69±0.03a	n.d	n.f	—	Peach, almond aroma
Methyl-4-decenoate	1620	n.d	n.d	n.d	n.d	n.d	n.d	5.28±0.40a	3	>1	n.f
Ethyl caprate	1645	1.46±0.05d	5.65±0.27a	1.82±1.10c	n.d	2.42±0.07b	1.88±0.08c	1.86±0.14c	200	<0.1	Coconut fragrance
Ethyl benzoate	1658	2.82±0.10f	4.35±0.21d	5.05±0.27c	3.35±0.03e	5.55±0.15b	6.53±0.30a	4.11±0.31d	60	<0.1	Winter oil, fruity
Ethyl-4-decenoate	1672	n.d	n.d	n.d	n.d	n.d	n.d	7.11±0.55a	n.f	—	Peach, floral
P-Tolyl acetate	1722	n.d	n.d	0.63±0.03a	n.d	n.d	n.d	n.d	n.f	—	Floral, lily, daffodils
Ethyl phenylacetate	1771	n.d	n.d	n.d	n.d	n.d	n.d	0.63±0.05a	250	<0.1	Honey
Phenethyl acetate	1808	6.99±0.25g	11.39±0.54f	26.43±1.39c	15.56±0.13e	29.54±0.82b	21.72±1.00d	44.42±3.41a	45.5	<0.1	Sweet, rose, peach
Phenylethyl octanoate	2373	n.d	n.d	n.d	n.d	n.d	0.60±0.03a	n.d	n.f	—	Fruity, fruit wine
**Lactones**											
γ-Caprolactone	1709	6.43±0.23d	18.91±0.90a	2.29±0.12g	5.23±0.04e	3.27±0.09f	13.23±0.61b	9.20±0.71c	260	<0.1	Herb, sweet
γ-Heptalactone	1784	n.d	1.53±0.07a	n.d	n.d	0.41±0.01c	0.64±0.03b	1.51±0.12a	400	<0.1	Nutty, caramel
γ-Octalactone	1886	0.85±0.03f	6.99±0.33b	2.34±0.12e	n.d	3.41±0.10d	6.35±0.29c	8.87±0.68a	6.5	0.1-1	Floral, sweet, fruity
δ-Octalactone	1967	n.d	0.51±0.02b	0.40±0.02c	0.28±0.01d	n.d	1.70±0.08a	n.d	n.f	—	Fruity, coconut
γ-Nonalactone	2008	n.d	n.d	0.32±0.02b	n.d	n.d	2.27±0.10a	n.d	9.7	0.1-1	Coconut, peach
γ-Decalactone	2109	6.14±0.22f	176.52±8.36a	18.31±0.96e	20.40±0.17e	31.65±0.88d	114.51±5.27b	84.26±6.47c	0.7	>1	Fruity, peach, creamy
δ-Decalactone	2193	1.42±0.05f	30.91±1.46a	2.76±0.14ef	3.71±0.03e	19.40±0.54c	16.72±0.77d	21.20±1.62b	31	0.1-1	Fruity, peach, creamy
γ-Dodecalactone	2376	n.d	1.46±0.07b	n.d	n.d	n.d	5.25±0.24a	n.d	0.43	>1	Peach
**Terpenes**											
β-Myrcene	1167	n.d	n.d	n.d	n.d	2.58±0.07a	n.d	n.d	100	<0.1	Fatty, sweet

Limonene	1205	n.d	n.d	n.d	n.d	5.74±0.16a	n.d	n.d	200	<0.1	Lemon
Styrene	1267	18.70±0.67b	32.52±1.54a	n.d	n.d	n.d	n.d	n.d	3.6	>1	Slightly sweet
Linalool	1556	n.d	212.03±10.04a	n.d	n.d	n.d	n.d	n.d	6	>1	Floral, green
**Others**											
Ethylbenzene	1153	11.96±0.43	n.d	n.d	n.d	5.35±0.15b	n.d	0.55±0.04c	n.f	—	Fragrance
2-Ethylfuran	945	n.d	n.d	33.71±1.77a	23.64±0.20b	n.d	14.34±0.66d	17.83±0.37c	100	0.1-1	Burned, sweet
2-Pentylfuran	1228	11.11±0.40a	5.05±0.24b	0.88±0.05c	n.d	n.d	n.d	n.d	6	>1	Green, fruity
Theaspirane	1523	n.d	n.d	0.49±0.03c	n.d	3.76±0.10b	n.d	4.42±0.34a	0.2	>1	Black tea, wood

[1] Mass spectrum and Kovats index agreed with literature data. [2] OT, Odor threshold.

Table S2. Concentration (%) of aroma compounds in seven peach cultivars.

Variety	YP	RN	LG	HP	ST	YN	FP
Alcohols	59.30	25.73	24.20	24.35	31.39	30.08	30.61
Acids	1.28	0.68	0.45	0.36	1.23	0.93	0.60
Aldehyde	20.64	31.76	63.02	64.43	49.89	43.83	39.72
Ketones	2.76	1.86	1.75	1.71	1.42	1.47	3.51
Ester	5.84	4.40	5.95	5.05	9.58	8.47	11.74
Lactones	2.60	17.18	1.85	2.12	5.24	13.93	11.19
Terpenes	3.28	17.74	—	—	0.75	—	—
Others	4.30	1.36	2.78	1.97	1.32	1.29	2.63

Supplementary Figures

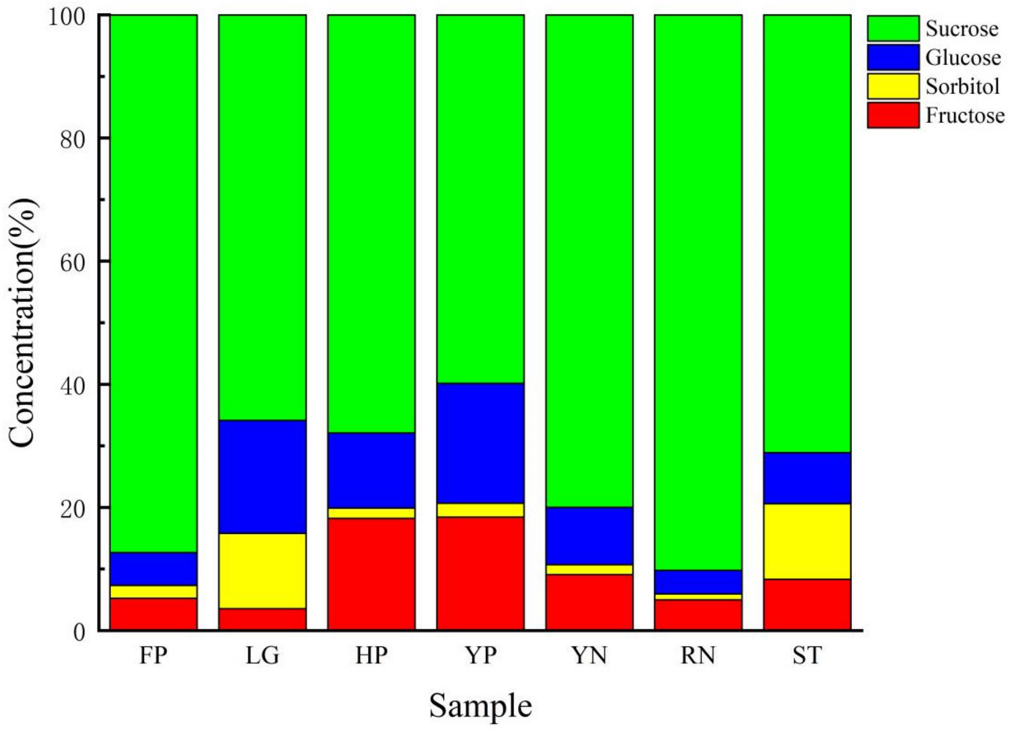

Figure S1. The relative concentration of sugar in seven peach cultivars.

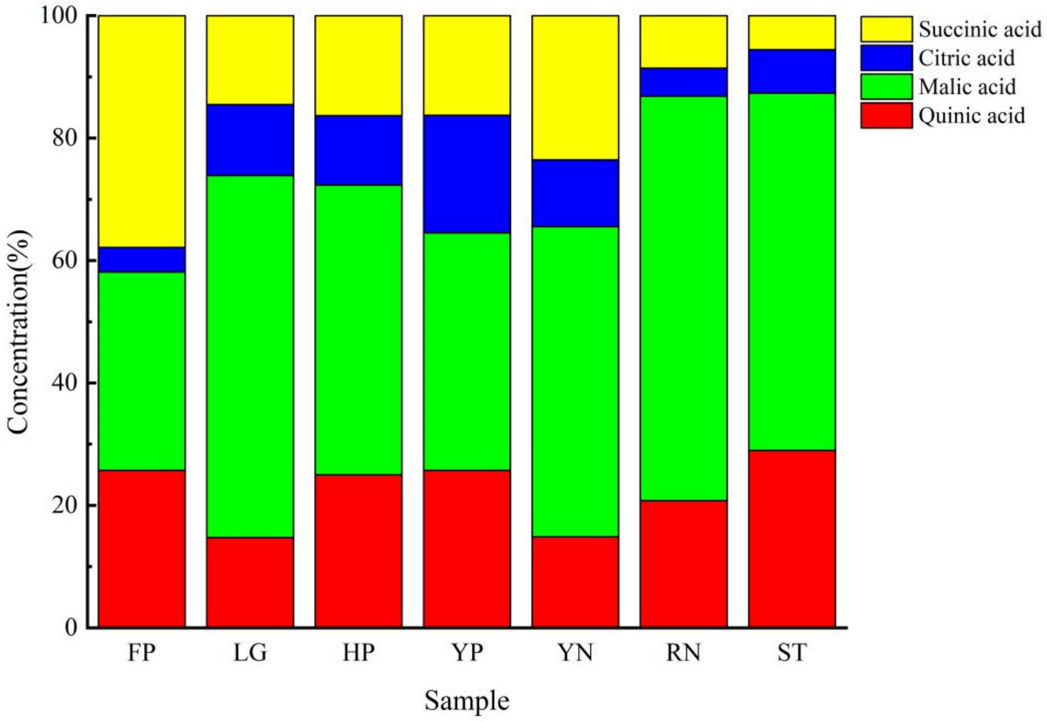

Figure S2. The relative concentration of organic acid in seven peach cultivars.

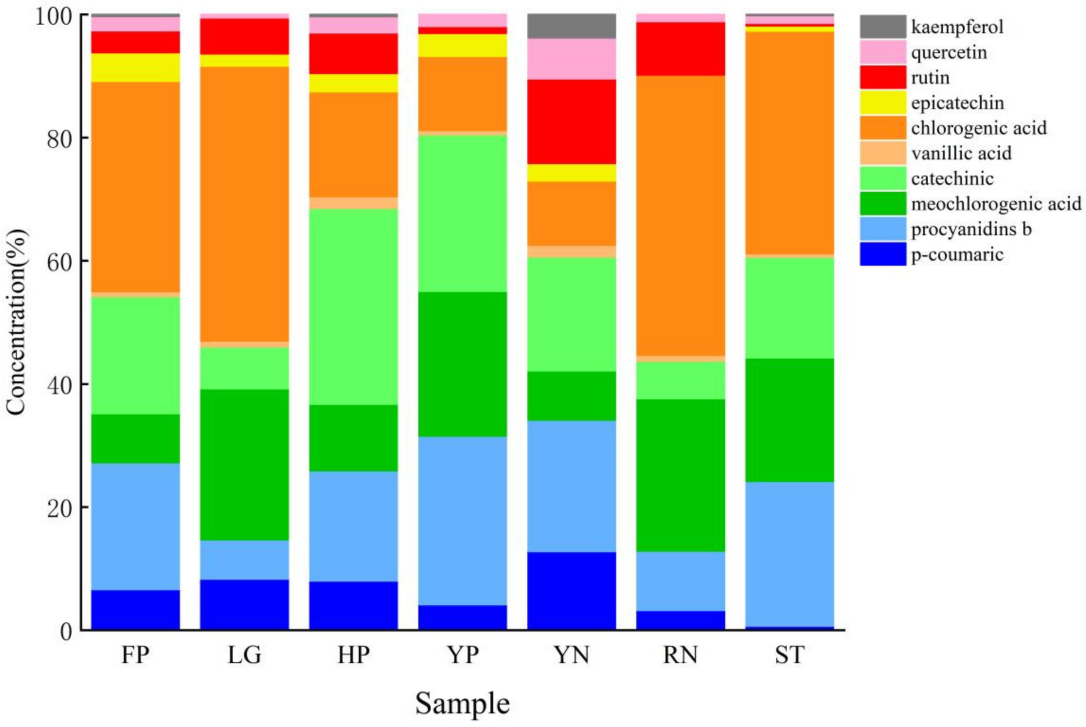

Figure S3. The relative concentration of phenolic compounds in seven peach cultivars.

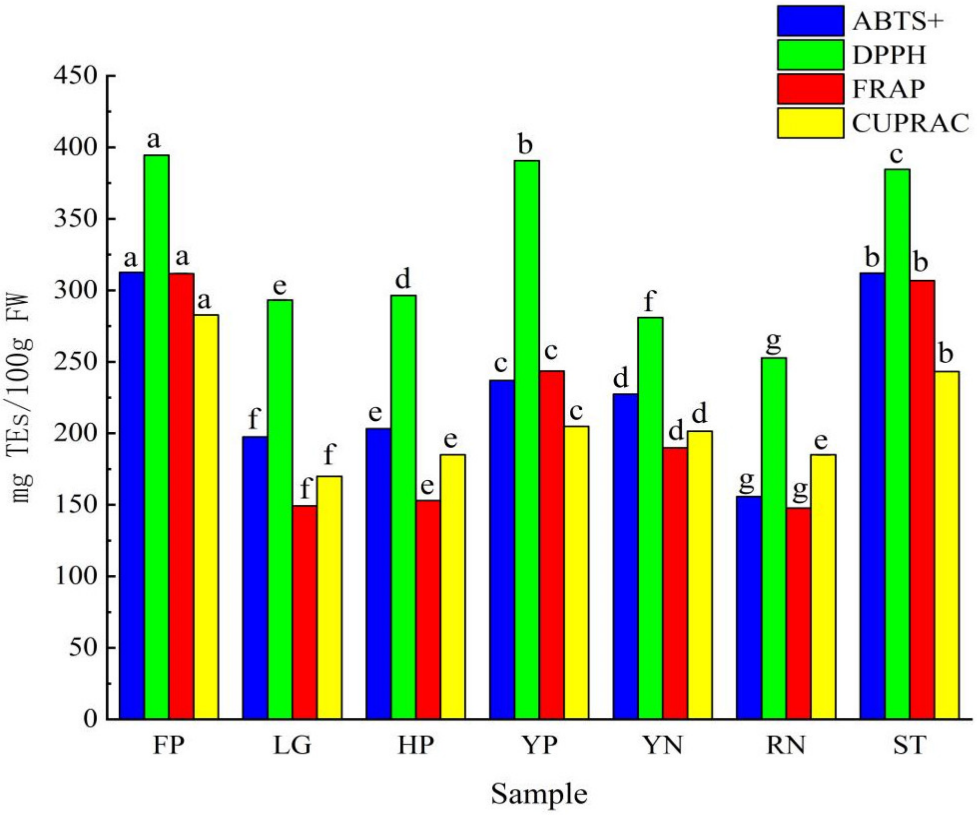

Figure S4. The antioxidant capacities in seven peach cultivars.

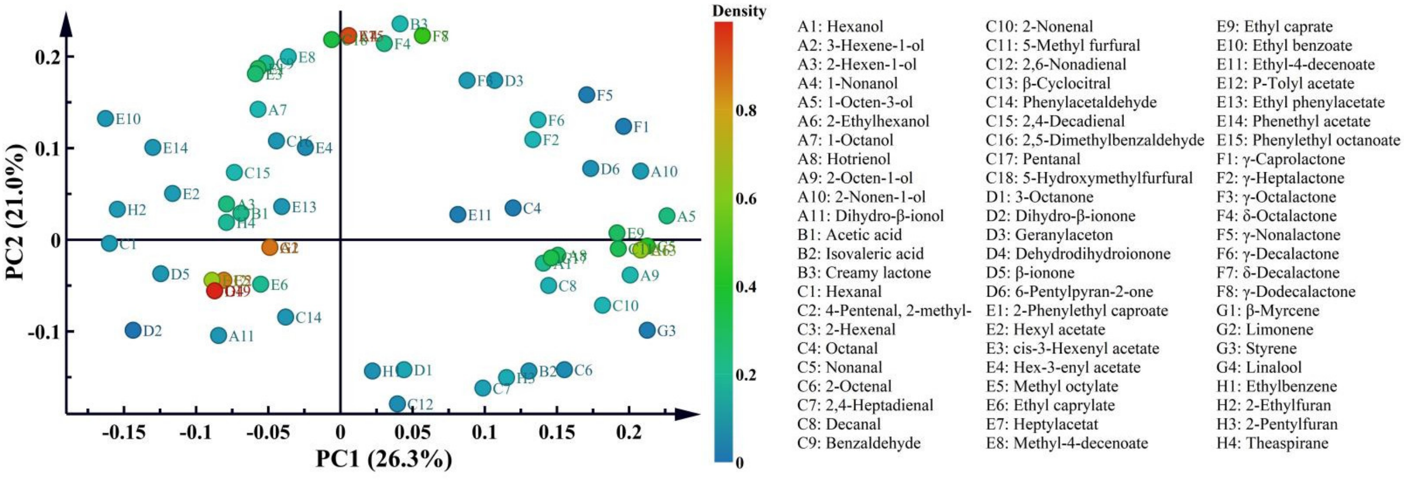

Figure S5. The loading diagram of aroma compounds in seven peach cultivars.

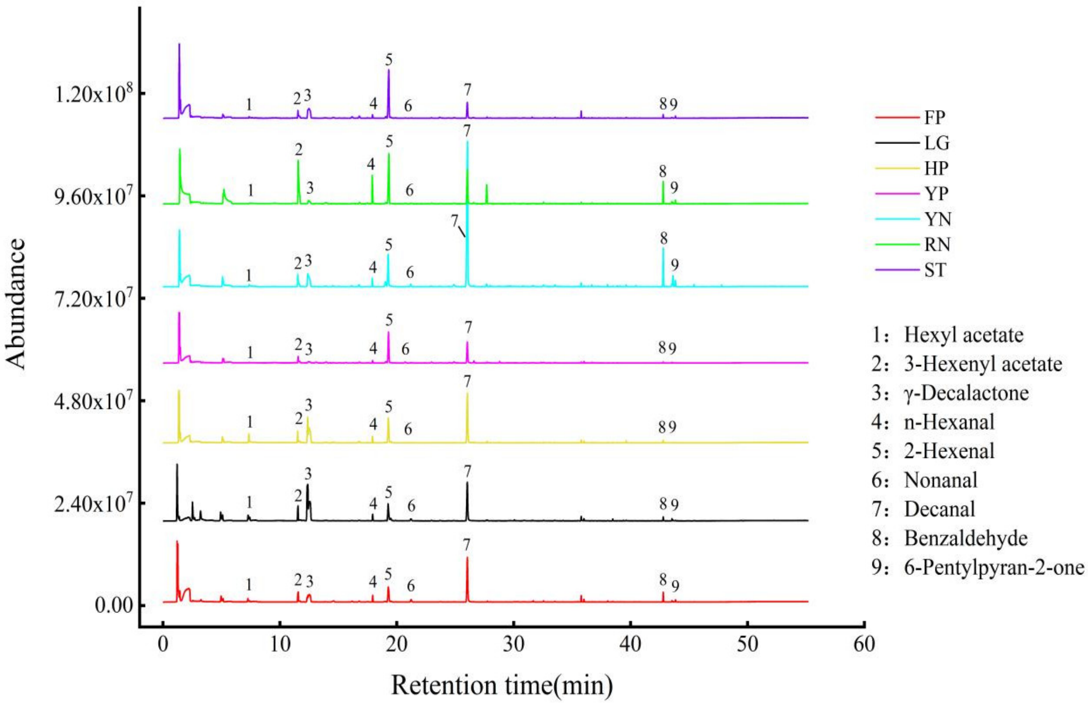

Figure S6. The **ion chromatograms of the seven peach cultivars**. The aroma substances with OAVs above 1 were marked.
